# Doxycycline-Induced Severe Thrombocytopenia: A Case Report

**DOI:** 10.7759/cureus.117542

**Published:** 2026-10-06

**Authors:** Benyam G Yirdaw, Rabia Mansoor, Umar Mahboob

**Affiliations:** 1 Acute Medicine/Respiratory Medicine, Fairfield General Hospital, Manchester, GBR

**Keywords:** doxycycline treatment, drug-induced thrombocytopenia, immune-mediated side effects, osteomyelitis treatment, platelet destruction

## Abstract

Doxycycline is a widely used antibiotic for commonly encountered infections and is generally well tolerated. However, it is associated with rare, drug-induced severe thrombocytopenia, which, if not promptly recognised and managed, could prove fatal. We present a case of doxycycline-induced severe thrombocytopenia in a 63-year-old man with a background of type 2 diabetes mellitus, left ventricular systolic dysfunction, and hypothyroidism. The platelet count dropped to a critical level of 6 x 10⁹/L following a one-week course of doxycycline, which improved with drug withdrawal, platelet transfusions, and a course of high-dose steroid therapy over three weeks. This was evidenced by a Naranjo Adverse Drug Reaction Probability Scale score of 8/13, which is the upper limit for a probable adverse drug reaction. This rare but life-threatening side effect of doxycycline highlights the need for a follow-up blood test shortly after commencing therapy for early detection and prompt management.

## Introduction

Thrombocytopenia is defined as a platelet count below 150 × 10⁹/L and is a common haematological abnormality in clinical practice [1]. Its causes can broadly be classified as reduced platelet production, increased peripheral destruction or consumption, splenic sequestration, or dilution. Important causes include infection, liver disease, malignancy, bone marrow disorders, immune thrombocytopenia (ITP), thrombotic microangiopathies, and medication-related thrombocytopenia [1,2].

Drug-induced thrombocytopenia (DITP) is an uncommon but important cause of acute thrombocytopenia. A wide range of medications has been implicated, including quinine, antibiotics, heparin, anticonvulsants, and antiplatelet agents [3,4]. DITP can result from several immune mechanisms, including drug-dependent antibodies, hapten-mediated reactions, drug-induced autoantibodies, and immune-complex formation [3,4]. Diagnosis is primarily clinical and requires assessment of the temporal relationship between drug exposure and platelet decline, exclusion of alternative causes, and, where available, demonstration of drug-dependent platelet antibodies. However, because such testing is specialised, not routinely available, and lacks standardisation, a negative or unavailable laboratory test does not necessarily exclude a clinically suspected diagnosis [3,4].

The mainstay of managing suspected DITP is prompt withdrawal of the offending medication. Platelet recovery generally begins after discontinuation, although the timing and degree of recovery may vary according to the responsible drug and individual patient factors [3,4]. Supportive management is guided by the severity of thrombocytopenia and the presence of clinically significant bleeding. Platelet transfusion may be considered in patients with profound thrombocytopenia or significant bleeding, and intravenous immunoglobulin or corticosteroids may be used in selected severe cases. Nevertheless, evidence supporting corticosteroid therapy specifically for DITP remains limited, and prompt recognition and withdrawal of the suspected causative medication remain central to management [3,4].

Doxycycline is a broad-spectrum, tetracycline-class antibiotic commonly used to treat a variety of bacterial infections. Although doxycycline is generally well tolerated, thrombocytopenia is listed among its reported haematological adverse reactions in the current UK Summary of Product Characteristics [5]. Clinically significant thrombocytopenia following doxycycline exposure appears to be uncommon, and there is limited published evidence describing this association. A recent report described two patients who developed severe thrombocytopenia shortly after commencing doxycycline and whose platelet counts recovered after the drug was withdrawn [6]. Given the relative scarcity of reported cases, clinicians may not immediately consider doxycycline when evaluating an abrupt and otherwise unexplained fall in platelet count, particularly in patients receiving treatment for complex infections or taking concomitant medications.

This case of profound thrombocytopenia shortly after doxycycline initiation adds to the limited published evidence of severe thrombocytopenia associated with doxycycline and highlights the importance of considering DITP when an abrupt and otherwise unexplained reduction in platelet count follows medication exposure.

## Case presentation

A 63-year-old man presented with a two-day history of feeling generally unwell. His medical history included type 2 diabetes mellitus, hypothyroidism, and left ventricular systolic dysfunction. He had been admitted to another hospital for a diabetic foot infection with features of osteomyelitis following recent corrective metatarsal surgery. He was given a one-week course of intravenous teicoplanin, followed by a six-week course of oral doxycycline, which he had been taking for about a week. On discharge, his blood tests showed a haemoglobin level of 111 g/L, a white cell count of 5.6 × 10⁹/L, a platelet count of 344 × 10⁹/L, and a C-reactive protein (CRP) level of 7.6 mg/L. Follow-up blood tests in the community, performed five days later, showed a haemoglobin level of 126 g/L, a white cell count of 5 × 10⁹/L, a platelet count of 289 × 10⁹/L, and a CRP level of 8 mg/L.

Apart from feeling generally unwell, he had no cardiovascular, respiratory, gastric, or urinary symptoms. He had no travel history. Physical examination was unremarkable. Blood tests revealed a full blood count with a haemoglobin level of 119 g/L, a white cell count of 3.8 × 10⁹/L, and a platelet count of 6 × 10⁹/L; a coagulation profile with a prothrombin time of 12.5 seconds, a normal international normalised ratio of 1.1, and an activated partial thromboplastin time of 26.1 seconds; and biochemistry showing a CRP level of 55 mg/L, normal renal function, normal liver enzymes, and normal total and unconjugated bilirubin levels. A blood film showed normal white cell morphology, no fragmented red cells, and confirmed genuine thrombocytopenia. The reticulocyte count was 56 × 10⁹/L with a reticulocyte percentage of 1.4%, and the haptoglobin level was 1.75 g/L; both were within their reference ranges. Immunology revealed a positive antinuclear antibody with a Ro/SS-A antibody index (AI) of > 8 and a Ro60/SS-A 60 AI of > 8. However, he had shown similar immunologic results in 2020, when he was referred to the rheumatology clinic with back pain, wrist pain, and triphasic Raynaud-like colour change of the hand without sicca symptoms or dry eyes. He was subsequently lost to follow-up during the COVID-19 pandemic.

A viral screen for parvovirus B19, hepatitis B and C, cytomegalovirus, Epstein-Barr virus, and HIV was negative. CT of the neck, thorax, abdomen, and pelvis showed no lymphadenopathy or overt malignancy, except for a stable subpleural nodule, present since 2022, which does not require further follow-up according to British Thoracic Society guidelines [7]. A myeloma screen showed normal serum electrophoresis, a normal serum free light chain (sFLC) ratio, immunoglobulin G of 15 g/L, immunoglobulin M of 2.18 g/L, and a raised immunoglobulin A of 6.5 g/L, with normal renal function and calcium levels.

With a working diagnosis of doxycycline-induced thrombocytopenia, the drug was withheld, and two units of platelets were transfused. In view of the rising CRP and following discussion with microbiologists, a stat dose of gentamicin was given, and teicoplanin was restarted. A post-transfusion full blood count, performed on the same day, showed a haemoglobin level of 100 g/L, a white cell count of 3.1 × 10⁹/L, and a platelet count of 22 × 10⁹/L. Two days later, the platelet count dropped to 11 × 10⁹/L with an otherwise similar full blood count profile. Following discussion with haematology, high-dose prednisolone, 60 mg once daily, was started with a plan for regular full blood count monitoring. Twenty-four hours later, the platelet count had risen to 28 × 10⁹/L with the same full blood count profile; six days after presentation, it had reached 42 × 10⁹/L. Subsequent results showed an overall increase in the platelet count, as summarised in Table 1, and a full blood count on discharge revealed a haemoglobin level of 111 g/L, a white cell count of 10.4 × 10⁹/L, and a platelet count of 146 × 10⁹/L.

**Table 1:** A Pattern of Platelet Count Changes in Response to Doxycycline Use, Withdrawal, and Immunosuppression This table displays changes in platelet count following a course of doxycycline, withdrawal of the medication, and platelet transfusions with high-dose steroid therapy. Days in the second column refer to the number of days before, during, and after the admission of the patient with the diagnosis of doxycycline-induced thrombocytopenia

Days	Timeline	Platelet Count	Reference Range
17^th ^Pre-admission Day	Previous Admission for Osteomyelitis	270 x 10^9^/L	150-450 x 10^9^/L
14^th ^Pre-admission Day		307 x 10^9^/L	150-450 x 10^9^/L
12^th ^Pre-admission Day	Blood Test on Discharge	344 x 10^9^/L	150-450 x 10^9^/L
7^th ^Pre-admission Day	Follow-up Test in the Community	289 x 10^9^/L	150-450 x 10^9^/L
1^st ^Day	Re-admission Blood Test in A&E	6 x 10^9^/L	150-450 x 10^9^/L
1^st ^Day	After a Unit of Platelets	11 x 10^9^/L	150-450 x 10^9^/L
1^st ^Day	Following the 2^nd^ Unit of Platelets	22 x 10^9^/L	150-450 x 10^9^/L
3^rd^Day	High-Dose Prednisolone Initiated	11 x 10^9^/L	150-450 x 10^9^/L
4^th^​​​Day		28 x 10^9^/L	150-450 x 10^9^/L
5^th ^Day		17 x 10^9^/L	150-450 x 10^9^/L
7^th^ Day		42 x 10^9^/L	150-450 x 10^9^/L
8^th^Day		45 x 10^9^/L	150-450 x 10^9^/L
9^th^ Day		74 x 10^9^/L	150-450 x 10^9^/L
10^th ^Day		110 x 10^9^/L	150-450 x 10^9^/L
14^th ^Day		68 x 10^9^/L	150-450 x 10^9^/L
15^th ^Day		93 x 10^9^/L	150-450 x 10^9^/L
16^th^​​​​​​​ Day	Weaning of Prednisolone Initiated	124 x 10^9^/L	150-450 x 10^9^/L
18^th^Day	Full Blood Count on Discharge	146 x 10^9^/L	150-450 x 10^9^/L
26^th ^Day	Follow-up Blood Test in The Community	247 x 10^9^/L	150-450 x 10^9^/L

Table 2 summarises the important laboratory investigations performed, along with the reference ranges for each test. These investigations were instrumental in narrowing the differential diagnoses considered in this case.

**Table 2:** Summary of Laboratory Tests Performed This table displays important blood tests performed with results and reference ranges. The days refer to the number of days since the admission of the patient with the diagnosis of doxycycline-induced thrombocytopenia

	Days	Laboratory Tests	Result	Reference Range
1	1^st ^Day	White Cell Count	3.8 × 10^9^/L	4-11 × 10^9^/L
2	1^st ^Day	Haemoglobin	119 g/L	135-175 g/L
3	1^st ^Day	Reticulocyte Count	56 × 10^9^/L	50-100 × 10^9^/L
4	1^st ^Day	Reticulocyte Percent	1.4%	0.5-2.5%
5	1^st ^​​​​​​​Day	Prothrombin Time	12.5 seconds	11-13.5 seconds
6	1^st ^​​​​​​​Day	Activated Partial Thromboplastin Time	26.1 seconds	25-35 seconds
7	1^st ^​​​​​​​Day	International Normalised Ratio	1.1	0.8-1.2
8	2^nd ^Day	C-reactive Protein	55 mg/L	< 5 mg/L
9	3^rd ^Day	Alanine Aminotransferase	48U/L	< 41 U/L
10	3^rd ^Day	Alkaline Phosphatase	76 U/L	30-130 U/L
11	3^rd ^Day	Total Bilirubin	11 umol/L	< 21 umol/L
12	3^rd ^Day	Unconjugated Bilirubin	7 umol/L	< 12 umol/L
13	3^rd ^Day	Haptoglobin	1.75 g/L	0.3-2.0 g/L
14	3^rd ^Day	Antinuclear Antibody	Positive	
15	3^rd ^Day	Ro/SS-A Antibody	> 8 Antibody Index	0-0.9 Antibody Index
16	7^th ^Day	Creatinine	82 umol/L	49-90 umol/L
17	7^th ^Day	Estimated Glomerular Filtration Rate	87 mL/min/1.73m²	> 90 mL/min/1.73m²
18	12^th ^Day	Hepatitis B and C	Negative	
19	12^th ^​​​​​​​Day	Cytomegalovirus	Negative	
20	12^th ^​​​​​​​Day	Epstein-Barr Virus	Negative	
21	12^th ^​​​​​​​Day	HIV	Negative	

In hospital, intravenous teicoplanin was restarted and continued for several days. Following discussion with the patient and the microbiology team, and although teicoplanin was not implicated in the thrombocytopenia, the patient opted to change antibiotics as a precaution, and vancomycin was initiated instead to complete the course. As the thrombocytopenia was resolving satisfactorily, prednisolone was tapered, and the patient was discharged with haematology follow-up arranged. A week later, a follow-up blood test revealed a haemoglobin level of 118 g/L, a white cell count of 8.7 × 10⁹/L, and a platelet count of 247 × 10⁹/L. Figure 1 shows the course of the thrombocytopenia and its response to drug withdrawal and the treatment administered to reverse the severe thrombocytopenia.

**Figure 1:**
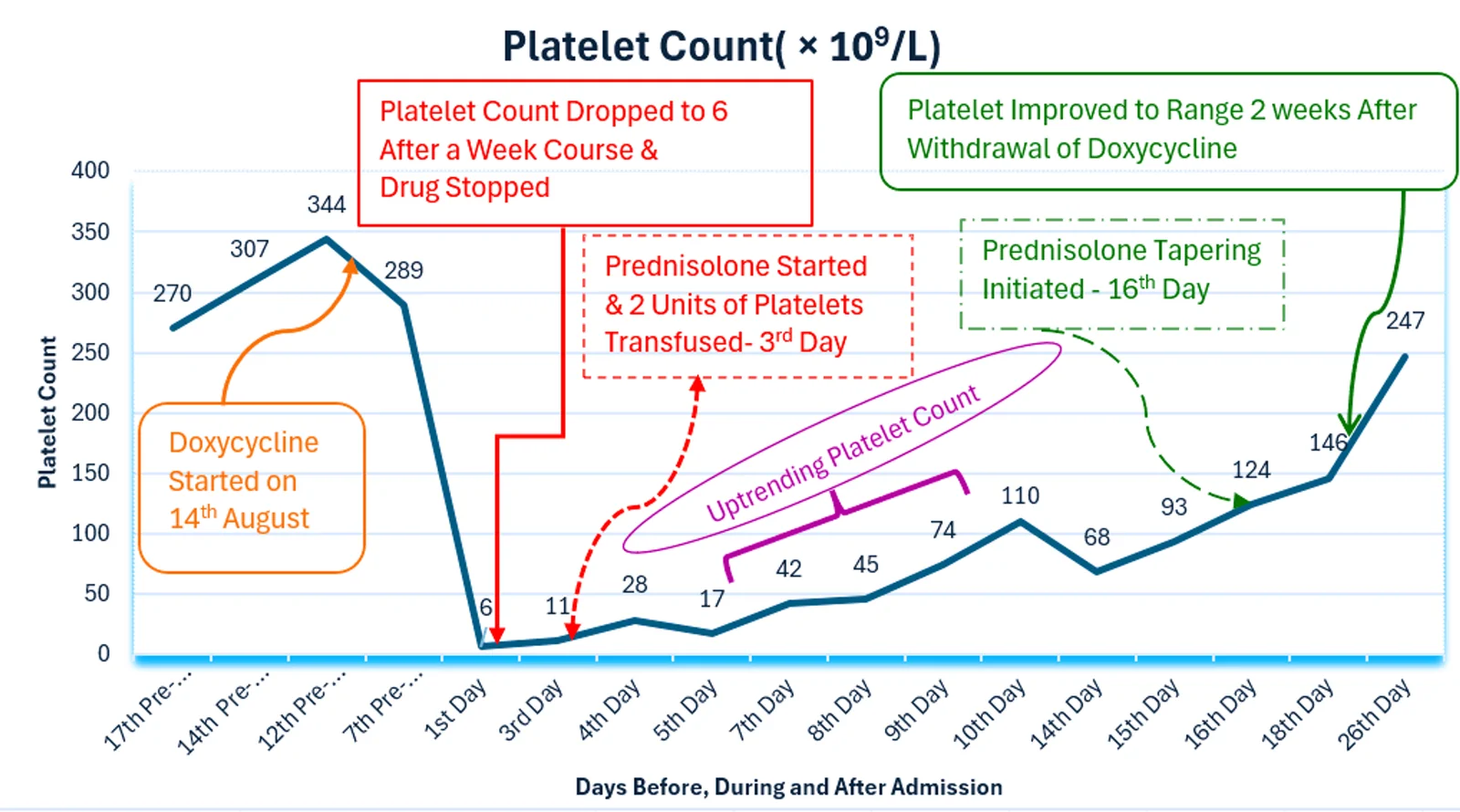
Platelet Count Mapped to Doxycycline Use, Withdrawal, and Therapy to Reverse Thrombocytopenia This line graph displays platelet counts in relation to the use of doxycycline, cessation of the drug, and platelet transfusions with the administration of high-dose prednisolone to reverse the severe thrombocytopenia. Platelet count dropped to a staggering level of 6 x 10^9^/L, which remained low for a week despite platelet transfusions and a course of high-dose steroid. Following this, the platelet count recovered to the normal range in two weeks' time The X-axis represents days before, during, and after the admission for doxycycline-induced thrombocytopenia. Pre-admission days refer to days before the admission of the patient with the diagnosis of drug-induced thrombocytopenia due to doxycycline

## Discussion

Doxycycline is a semisynthetic tetracycline antibiotic with bacteriostatic activity against Gram-positive and Gram-negative bacteria and is commonly used to treat a variety of infections, including respiratory, skin, and urogenital infections [6,8]. Its reported side effects include angioedema, diarrhoea, headache, Henoch-Schönlein purpura, hypersensitivity, nausea, pericarditis, photosensitivity reactions, skin reactions, exacerbation of systemic lupus erythematosus, and vomiting [8]. By contrast, the British National Formulary lists thrombocytopenia as a rare or very rare side effect of doxycycline [8]. Reports of doxycycline-induced thrombocytopenia are scant, based on our review and a systematic search of the EMBASE, Cochrane, PubMed, WanFang, and China National Knowledge Infrastructure databases using the keyword “doxycycline adverse reactions” [6]. The mechanism by which doxycycline causes thrombocytopenia has not been established, likely because so few cases have been reported; however, immune-mediated platelet destruction has been implicated in the reported cases [6,9].

DITP is a well-recognised phenomenon, and many medications have been reported to cause it, including penicillin, piperacillin, cephalosporin antibiotics, quinine, non-steroidal anti-inflammatory drugs, anticonvulsants, eptifibatide, tirofiban, abciximab, gold salts, L-dopa, procainamide, and heparin [3,10]. Several immunologic mechanisms by which drugs lead to antibody-mediated platelet destruction have been described, including hapten-mediated antibody formation, drug-dependent antibody production, autoantibody induction, and immune-complex formation [3,10]. However, published reports of doxycycline-induced thrombocytopenia and its pathogenesis remain scarce.

This report describes a case of doxycycline-induced thrombocytopenia that resolved with drug discontinuation, platelet transfusion, and steroid therapy. Our patient was initially treated with teicoplanin for osteomyelitis for seven days, followed by a planned six-week course of doxycycline. However, he re-presented with a platelet count of 6 × 10⁹/L after more than a week of antibiotic treatment. Full blood counts taken in hospital after the course of teicoplanin and again five days later in the community revealed no thrombocytopenia, making teicoplanin an unlikely culprit. Moreover, the platelet count continued to improve despite several days of teicoplanin after it was restarted, further supporting the absence of a temporal association between teicoplanin and the fall in platelet count. In contrast, the platelet count was critically low on admission after a course of doxycycline and improved once the drug was withdrawn, with the subsequent recovery likely reflecting the combined effects of platelet transfusions, steroids, and drug cessation. This temporal association between doxycycline and thrombocytopenia supports the working diagnosis of doxycycline-induced thrombocytopenia.

Possible differential diagnoses for the thrombocytopenia that were considered but ruled out included thrombotic thrombocytopenic purpura (TTP), ITP, infective causes, and malignancies. The patient’s haemoglobin level was relatively stable, and reticulocyte, bilirubin, and haptoglobin levels were normal; blood films were suggestive of genuine thrombocytopenia without polychromasia, schistocytes, or spherocytes. Moreover, the patient did not have renal, neurologic, or cutaneous signs, and there was no evidence of microangiopathic haemolysis, making TTP unlikely. The absence of a sustained isolated thrombocytopenia pattern or other features suggestive of ITP was also considered in the overall clinical assessment.

Infection, although a possible cause of a low platelet count, was considered less likely based on the negative viral screen results. CT of the neck, thorax, abdomen, and pelvis revealed no lymphadenopathy or malignancy and, together with relatively stable full blood count results apart from a persistent mild anaemia and the acute thrombocytopenia, made an underlying malignancy unlikely as a cause. Another differential, considered unlikely, was myeloma, as serum electrophoresis, sFLC, renal function, and calcium levels were normal. Furthermore, the patient denied bone pain, and CT of the neck, thorax, abdomen, and pelvis was not suggestive of myeloma.

This leaves doxycycline-induced thrombocytopenia as the probable cause of the presentation, given the close temporal association between doxycycline initiation and the onset of thrombocytopenia, and its resolution after drug withdrawal. This is further supported by the Naranjo Adverse Drug Reaction Probability Scale score of 8/13, which falls within the probable category for doxycycline-induced thrombocytopenia [11]. On this scale, a probable score means that the reaction followed a reasonable temporal sequence after drug administration, fitted a recognised pattern of response to the suspected medication, and was supported by improvement on withdrawal of the drug [11]. In our patient, severe thrombocytopenia developed after a course of doxycycline and improved with drug withdrawal, platelet transfusion, and a short course of high-dose steroids.

A systematic search of bibliographic databases, including EMBASE, Cochrane, and PubMed, reported in a case report publication [6], found only a single report of doxycycline-induced thrombocytopenia, indexed in EMBASE in 2009 [12]. The same publication presented and discussed two additional cases of doxycycline-induced thrombocytopenia [6].

One case report, published in 2026, described severe thrombocytopenia after a one-week course of doxycycline in a patient with tsutsugamushi disease [6]. The platelet count dropped to 3 × 10⁹/L and reached a nadir of 1 × 10⁹/L despite transfusion of two units of platelets. With a suspected diagnosis of doxycycline-induced thrombocytopenia, further transfusions, along with human interleukin-11 and thrombopoietin receptor agonists, were given. This was followed by pulse therapy with intravenous immunoglobulin at 0.5 g/kg and avatrombopag, given the lack of response. High-dose methylprednisolone was then added to the high-dose immunoglobulin pulse therapy. The patient’s platelet count began to rise after two weeks of combined immunotherapy and normalised 24 hours later. Our case is similar to this one in that severe thrombocytopenia (platelet count 6 × 10⁹/L) developed after a short course of doxycycline and resolved following the combined effects of drug withdrawal and immunotherapy. However, the presence of bleeding at presentation and the use of intensive immunotherapy with intravenous immunoglobulin, human interleukin-11, thrombopoietin receptor agonists, and avatrombopag before platelet recovery distinguish that case from ours.

The second case in that publication involved a 75-year-old patient admitted with sepsis, with Salmonella confirmed on blood culture and foci of chest and intra-abdominal infection, who was started on imipenem-cilastatin (500 mg every four hours via an infusion pump) and moxifloxacin (250 mL once daily intravenously) [6]. A repeat culture revealed Salmonella group D, and CT of the abdomen showed blurring of the abdominal wall, following which doxycycline was initiated to cover Gram-positive and Gram-negative pathogens because peritonitis was clinically suspected. After two days of doxycycline treatment, blood results showed a precipitous drop in the platelet count to 2 × 10⁹/L. With a suspected diagnosis of doxycycline-induced thrombocytopenia, the drug was withdrawn, platelet transfusions were given, and pulse therapy with high-dose prednisolone and immunoglobulin was initiated. The platelet count improved to over 50 × 10⁹/L within the same week as the pulse therapy and normalised after two weeks. The sepsis was effectively treated with antibiotic optimisation and antifungal coverage, and the patient was discharged with blood test results within normal ranges. This case followed a similar pattern to ours and to the case discussed above, in that thrombocytopenia developed after doxycycline initiation and improved with drug cessation and immunotherapy.

These cases highlight the sudden and precipitous drop in platelet count that can follow a short course of doxycycline, with potentially life-threatening complications, such as intracranial haemorrhage. In light of this, consideration should be given to monitoring platelet counts after starting doxycycline, even though the British National Formulary [8] lists this side effect as rare or very rare. A high index of suspicion, prompt withdrawal of the drug, and appropriate treatment with corticosteroids and, where indicated, intravenous immunoglobulin are important in managing severe cases and preventing life-threatening complications such as intracranial haemorrhage.

## Conclusions

Doxycycline is a broad-spectrum antibiotic used to treat various infections, such as respiratory, urogenital, and cutaneous infections, and is active against Gram-positive, Gram-negative, and atypical bacteria. However, severe thrombocytopenia, although rare, is a potentially life-threatening adverse effect of doxycycline therapy. We describe a case of severe thrombocytopenia following a short course of doxycycline therapy. Given the sudden and precipitous fall in platelet count and the potential risk of life-threatening bleeding, clinicians should consider monitoring the full blood count within a few days of initiating doxycycline.
